# Elevated Natural Killer Cell-Mediated Cytotoxicity Is Associated with Cavity Formation in Pulmonary Tuberculosis Patients

**DOI:** 10.1155/2021/7925903

**Published:** 2021-10-04

**Authors:** Shanshan Li, Dongpo Wang, Panjian Wei, Rongmei Liu, Jidong Guo, Bin Yang, Hongtao Zhang, Jie Lu, Mengqiu Gao, Yu Pang

**Affiliations:** ^1^Department of Bacteriology and Immunology, Beijing Chest Hospital, Capital Medical University/Beijing Tuberculosis & Thoracic Tumor Research Institute, Beijing 101149, China; ^2^Department of Radiology, Beijing Chest Hospital, Capital Medical University/Beijing Tuberculosis & Thoracic Tumor Research Institute, Beijing 101149, China; ^3^Department of Central Laboratory, Beijing Chest Hospital, Capital Medical University/Beijing Tuberculosis & Thoracic Tumor Research Institute, Beijing 101149, China; ^4^Department of Tuberculosis, Beijing Chest Hospital, Capital Medical University/Beijing Tuberculosis and Thoracic Tumor Research Institute, Beijing 101149, China; ^5^Beijing Key Laboratory for Pediatric Diseases of Otolaryngology, Head and Neck Surgery, MOE Key Laboratory of Major Diseases in Children, Beijing Pediatric Research Institute, Beijing Children's Hospital, Capital Medical University, National Center for Children's Health, Beijing 101149, China

## Abstract

Cavitation is a major pathological feature of pulmonary tuberculosis (TB). The study is aimed at investigating the mechanism of natural killer (NK) cells participating the cavity formation during *Mycobacterium tuberculosis* (MTB) infection. Human peripheral blood samples were donated by pulmonary TB patients with cavity or not. Real-time quantitative PCR and enzyme-linked immunosorbent assay were performed to analyze the expression of cytokines secreted by NK cells. And the cytotoxicity of NK cells was compared between two groups. Our data showed that NK cells were more abundant in cohorts of cavity. Increased abundance of granzyme A and granzyme B was observed in culture supernatants of NK cells isolated from cavitary TB patients, which also resulted in a higher level of nonviable MTB-infected monocytes. Our data firstly demonstrates that NK cells participate in cavity formation in pulmonary TB patients. The elevated level and increased cytotoxicity of NK cells accelerate the cavitary formulation.

## 1. Introduction

Tuberculosis (TB), caused by *Mycobacterium tuberculosis* (MTB) complex, is currently the leading cause of death from a single infectious pathogen [1]. In 2018, this disease was responsible for an estimated 10.0 million incident cases and 1.4 million deaths worldwide [1]. Cavitation is a major pathological feature of human pulmonary TB and is the site of very large bacillary burden [2, 3]. The presence of pulmonary cavitation is associated with poor treatment outcomes, such as prolonged sputum culture conversion, relapse after treatment, and acquisition of drug resistance [4, 5]. In addition, the patients with pulmonary cavity are more infectious than those with noncavitary disease, recognized as a principle source for TB transmission in the community [2, 6]. The contribution of cavitation to the increased risk of person-to-person TB transmission makes it an important target for therapeutic invention [7].

Evidence from clinical cohorts demonstrated that the rates of cavitary tuberculosis at the time of diagnosis range from 29% to 87% [8–10]. A better understanding of the pathogenesis of cavity formation is of great significance in reduction of adverse outcome for pulmonary TB patients and prevention of TB transmission. There is a widely held view that cavitation is originated from granulomas coalesce and break down via liquefactive necrosis [11, 12]. Thus, the complex interaction between tubercle bacilli and host immunity constitutes the underlying mechanisms for cavity formation. In this aspect, the uncontrolled immune response against MTB leads to clearance of MTB-infected cells, accompanied by accelerated tissue and organ damage. Previous studies demonstrated that the T-helper-2 cytokine adaptive immune response predominated in cavitary TB patients [13, 14], indicating that the attenuation of the host-protective regulatory T cell response reduces their ability to control multiplication of MTB and leads to cavity formation. However, investigators still debate the directionality of this association, given that pulmonary TB patients with cavitary disease had higher amounts of the proinflammatory cytokines in bronchoalveolar lavage, such as tumor necrosis factor- (TNF-) *α*, interleukin- (IL-) 6, and IL-1*β* [15, 16]. Therefore, an alternative hypothesis proposes that the cavitation is the result of a progressive immune response to MTB despite little is known.

Natural killer (NK) cells are cytotoxic innate lymphocytes that produce high levels of proinflammatory cytokines in response to pathogens [17]. These cells have the capability to recognize and induce apoptosis in target cells via the release of perforin and granzymes [18]. Based on the existing data, both circulating and tissue-resident NK cells contribute to host protection, as well as possible adverse effects in the tissue. In view of the remarkable cytotoxic activity of NK cells, the question is raised as to whether their biased inflammatory response correlates with cavity diseases. The study is aimed at investigating the mechanism of NK cells participating the cavity formation during MTB infection.

## 2. Materials and Methods

### 2.1. Patients and Ethics Statement

Human peripheral blood samples were donated by pulmonary TB patients with cavity or not and healthy uninfected donors. All pulmonary TB patients accepted primary treatment. The subjects with other acute or chronic diseases, including diabetes, hepatitis, tumor, and immune dysfunction, were excluded for analysis. Pulmonary TB patients were defined with positive sputum smears, cultures, and/or GeneXpert MTB/RIF (Cepheid, USA) for *Mycobacterium tuberculosis* with pulmonary imaging features. Fourteen pulmonary TB patients with cavity and eleven pulmonary TB patients with noncavity were recruited to analyze the cytokines secreted by NK cells after cultivation with MTB H37Rv protein lysates. In addition, nine pulmonary TB patients with cavity and eight pulmonary TB patients with noncavity were recruited for NK cell cytotoxicity assay.

The information of all individuals involved in the study was anonymized. Written informed consent was obtained from all individuals. All procedures performed on human samples were carried out according to the tenets of the World Medical Association's Declaration of Helsinki and approved by the Ethical Committee of Beijing Tuberculosis and Thoracic Tumor Institute.

### 2.2. Analysis of Peripheral Lymphocyte Subsets

Peripheral blood samples were collected by venous puncture using vacutainer tubes containing ethylenediaminetetraacetic acid (EDTA) as anticoagulant. Flow cytometric analysis of peripheral lymphocyte subsets was performed by LSRII® flow cytometer (BD Biosciences, San Diego, CA, USA) using the BD FACSDiva software with the following monoclonal antibodies: BD Multitest™ 6-color TBNK reagent including CD3 FITC, CD16 PE, CD56 PE, CD45 PerCP-Cy™5.5, CD4 PE-Cy™7, CD19 APC and CD8 APC Cy™7 (BD Biosciences, CA, USA), BD Horizon™ BV510 anti-human CD127 clone HIL-7R-M21, BV421 mouse anti-human CD25clone M-A251 (BD Biosciences, CA, USA), anti-CD14-Violet 650 clone M5E2, and anti-human HLA-DR-Violet 605 clone L243 (Biolegend, San Diego, CA, USA). Lymphocyte subpopulations were characterized by combinations of surface markers as follows: CD3+ and CD45+ for T lymphocytes; CD3+, CD45+, and CD4+ for helper T cells; CD3+, CD45+, and CD8+ for cytotoxic T cells; CD4+, CD25+, and CD127low for regulatory T cells; CD45+ and CD19+ for B lymphocytes; CD45+, CD56+, and CD3- for NK cells; and CD3+, CD56+, and CD45+ for NK T cells. Data analysis was performed using the BD FACSDiva software (version 8.0.2).

### 2.3. Isolation and Culture of NK Cells

Peripheral blood mononuclear cells (PBMCs) were isolated from peripheral blood samples of pulmonary TB patients with cavity or not by density gradient centrifugation using lymphocyte separation medium (TBD, Tianjin, China). NK cells were isolated with human NK cell isolation kit (Miltenyi Biotec, Bergisch Gladbach, Germany) according to the manufacturer's instructions, and negatively selected cells were >95% CD16+/CD56+ cells as measured by flow cytometer. Isolated NK cells were plated in 24-well plates (Corning, USA) at 2 × 10^5^/well in 500 *μ*l of RPMI 1640 medium (HyClone, Waltham, USA) containing 10% fetal bovine serum (Gibco, USA) and incubated with 50 *μ*g/ml MTB H37Rv (ATCC 27294) protein lysates for 24 h at 37°C in a humidified 5% CO_2_ atmosphere.

### 2.4. Real-Time Quantitative PCR (RT-qPCR)

RT-qPCR was performed to analyze the mRNA expression of cytokines in cultured NK cells of both pulmonary TB patients with cavity or not. Total RNA was extracted from 2 × 10^5^ cultured NK cells using TRIzol reagent (Thermo Fisher Scientific, USA) according to the manufacturer's instructions. And RNA was treated with TURBO DNA-free™ kit (Thermo Fisher Scientific, USA) to remove contaminating DNA and was reverse transcribed to cDNA using Hifair II 1st strand cDNA synthesis supermix (Yeasen Biotech, Beijing, China). Then, real-time PCR assays were performed using Hifair® qPCR SYBR green master mix (No Rox) (Yeasen Biotech) on Roche LightCycler®480 II platform as follows: 95°C for 5 min and 40 cycles of 95°C for 10 s, 55°C for 30 s, and 72°C for 30 s. The primers for tumor necrosis factor-*α* (TNF-*α*) (forward, 5′-CCTCTCTCTAATCAGCCCTCTG-3′ and reverse, 5′-GAGGACCTGGGAGTAGATGAG-3′), natural cytotoxicity triggering receptor 1 (NKp46) (forward, 5′-TGGACCCGAAGTGATCTCG-3′ and reverse, 5′-TCCTTGAGCAGTAAGAACATGC-3′), granulysin (forward, 5′-CCTGTCTGACGATAGTCCAAAAA-3′ and reverse, 5′-GACCTCCCCGTCCTACACA-3′), perforin (forward, 5′-GGCTGGACGTGACTCCTAAG-3′ and reverse, 5′-CTGGGTGGAGGCGTTGAAG-3′), granzyme A (forward, 5′-TCTCTCTCAGTTGTCGTTTCTCT-3′ and reverse, 5′-GCAGTCAACACCCAGTCTTTTG-3′), and granzyme B (forward, 5′-CCCTGGGAAAACACTCACACA-3′ and reverse, 5′-GCACAACTCAATGGTACTGTCG-3′) were designed by Primer-BLAST (https://www.ncbi.nlm.nih.gov/tools/primer-blast). *β*-Actin was used as internal control, and the primers of which were 5′-CATGTACGTTGCTATCCAGGC-3′ (forward) and 5′-CTCCTTAATGTCACGCACGAT-3′ (reverse). The relative expression levels of each genes were analyzed using the 2^-*Δ*Ct^ method as previously described [19].

### 2.5. Enzyme-Linked Immunosorbent Assay (ELISA)

Due to the results of RT-qPCR, culture supernatants of NK cells of pulmonary TB patients with cavity or not were collected after 24 h cultivation to measure cytokine concentrations. For measurement of granzyme A and granzyme B, human granzyme A ELISA kit (RayBiotech, USA) and human granzyme B ELISA kit (RayBiotech, USA) were used according to the manufacturer's instructions. And optical density (OD) values at 450 nm were read on a Multiskan FC microplate photometer (Thermo Fisher Scientific, USA).

### 2.6. Isolation of Monocytes and MTB Infection

Monocytes collected from one healthy donor were isolates using human pan monocyte isolation kit (Miltenyi Biotec, Germany) and plated in 6-well plates (Corning, USA) in 2 ml RPMI 1640 medium (HyClone) containing 10% fetal bovine serum (Gibco, USA). Then, cells were infected with MTB H37Rv at a MOI of 2 : 1 and incubated for 2 h at 37°C in 5% CO_2_ atmosphere. After incubation, cells were washed with PBS to remove extracellular tubercle bacillus.

### 2.7. Flow Cytometry Analysis for NK Cell Cytotoxicity Assay

NK cells were collected and treated with MTB H37Rv protein lysates as described above. And infected monocytes were cocultured with NK cells (2 × 10^5^/well) at a ratio of 1 : 2 at 37°C in a humidified 5% CO_2_ atmosphere. After 4 h cultivation, cells were collected and incubated with specific antibodies at manufacturer's recommended concentrations at 4°C for 30 min to analyze the percentage of nonviable CD14+ monocytes by flow cytometry. The antibodies used in the study were as follows: PE anti-human CD56, brilliant violet 510™ anti-human CD16, APC anti-human CD14, and brilliant violet 711™ anti-human CD3 (Biolegend, San Diego, CA, USA). And fixability viability stain 440UV (FVS440UV, BD Biosciences, USA) was used for discrimination of nonviable from viable CD14+ monocytes to measure NK cell cytotoxicity. The cells were detected using BD LSRFortessa™ Flow Cytometer (BD Biosciences, USA), and results were analyzed using the BD FACSDiva software (version 8.0.2).

### 2.8. Statistical Analysis

To explore the relationship between pulmonary cavity or not with age, sex, ethic group, and treatment history, univariate analysis using the chi-square test was calculated. Results are shown as the mean ± standard deviations (SD). For data that were normally distributed, comparisons were performed by one-way ANOVA or unpaired Student's *t*-test. For data that were not normally distributed, the nonparametric Kruskal-Wallis test or Mann–Whitney *U* test was used. Values of *P* < 0.05 were considered statistically significant. The analyses were performed by the GraphPad Prism software version 6.0 and IBM's Statistical Package for the Social Sciences (SPSS) version 26.0.

## 3. Results

### 3.1. Peripheral Lymphocyte Subsets

**Figure 1:**
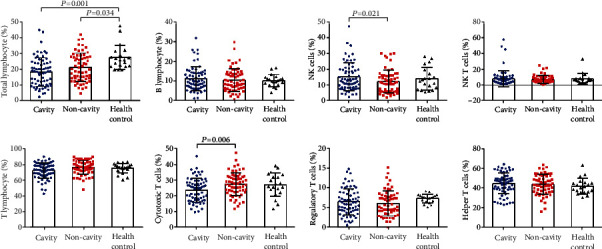
The proportion of peripheral lymphocytes in pulmonary TB patients with cavity or noncavity and healthy uninfected donors from our cohort. Comparisons for total lymphocytes (%), B lymphocytes (%), NK T cells (%), T lymphocytes (%), regulatory T cells (%), and helper T cells (%) were performed using the Kruskal-Wallis test. Comparisons for NK cells (%) and cytotoxic T cells (%) were performed using one-way ANOVA with least significance difference (LSD) multiple comparisons test due to normal distribution and homogeneity of variance.

To gain a comprehensive understanding of the role of immune cells in pulmonary cavity formation after MTB infection, sixty-eight pulmonary TB patients with cavity, sixty-one pulmonary TB patients with noncavity, and twenty healthy uninfected donors were recruited at Beijing Chest Hospital. The characteristics of subjects are summarized in Table 1. The results of flow cytometry analysis showed that there were no significant differences in B lymphocytes (11.17 ± 6.00, 10.51 ± 5.74 versus 10.33 ± 3.01), NK T cells (7.89 ± 10.36, 7.03 ± 4.88 versus 7.83 ± 6.96), T lymphocytes (72.22 ± 9.60, 76.08 ± 8.62 versus 75.07 ± 6.22), regulatory T cells (6.56 ± 3.30, 6.17 ± 3.16 versus 7.38 ± 1.07), and helper T cells (44.70 ± 10.42, 43.78 ± 10.03 versus 42.05 ± 7.89) among pulmonary cavitary TB patients, pulmonary noncavitary TB patients, and healthy uninfected donors (Figure 1). Total lymphocytes were significantly decreased in pulmonary cavitary TB patients (18.32 ± 9.09, *P* = 0.001) and pulmonary noncavitary TB patients (21.29 ± 8.95, *P* = 0.034) compared to uninfected controls (27.48 ± 7.65). And NK cells were significantly more abundant in cohorts of cavity compared to noncavity (15.27 ± 8.73 versus 12.20 ± 7.28, *P* = 0.021). However, analysis indicated a significant decrease in the percentages of cytotoxic T cells (23.92 ± 7.44 versus 27.53 ± 7.26, *P* = 0.006) in pulmonary cavitary TB patients compared to pulmonary noncavitary TB patients.

### 3.2. Analysis of Cytokines Secreted by NK Cells

**Figure 2:**
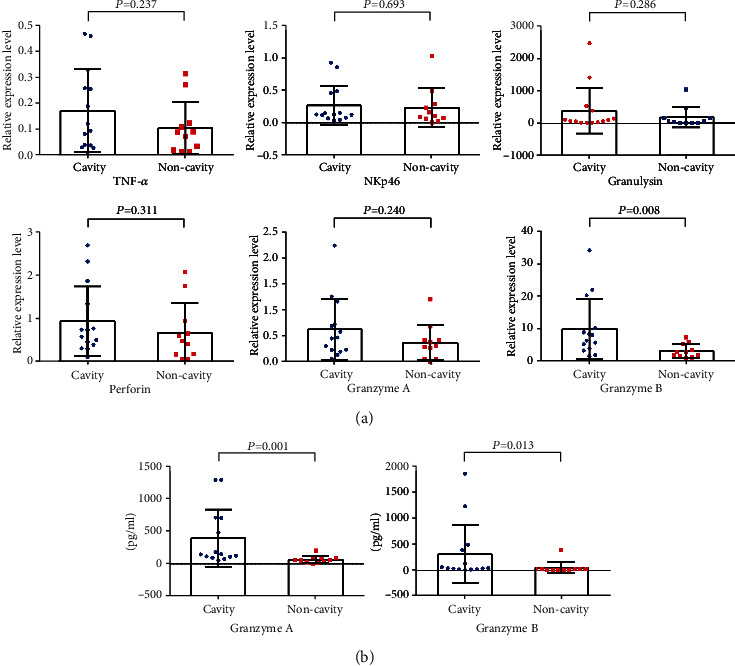
The expression levels of cytokines produced by NK cells from pulmonary TB patients with cavity or noncavity cultured with MTB H37Rv protein lysates. NK cells from fourteen pulmonary TB patients with cavity and eleven pulmonary TB patients with noncavity were isolated and cocultured with MTB H37Rv protein lysates to analyze the expression level of cytokines by (a) RT-qPCR and (b) ELISA. Throughout, *P* values were derived using a Mann–Whitney *U* test except TNF-*α*. For TNF-*α*, the *P* value was derived using unpaired Student's *t*-test due to normal distribution and homogeneity of variance.

To evaluated whether and how cytokines secreted by NK cells participate pulmonary cavitation in TB progression, we analyzed mRNA expression levels of TNF-*α*, interferon gamma (IFN-*γ*), interleukin 12 (IL-12), IL-22, granulocyte-macrophage colony-stimulating factor (GM-CSF), NKp30, NKp44, NKp46, granulysin, perforin, granzyme A, and granzyme B in NK cells by RT-qPCR (Figure 2(a)). Analysis indicated that granzyme B was increased significantly in pulmonary TB patients with cavity. And no significant differences in TNF-*α*, NKp46, granulysin, perforin, and granzyme A were observed between two groups of samples. Due to the expression level of IFN-*γ*, IL-12, IL-22, GM-CSF, NKp30, and NKp44 were lower than detection limitation in some RNA samples, data are not shown here. Then, we evaluated the cytokine concentrations secreted by NK cells (Figure 2(b)) as demonstrated by the results of RT-qPCR. Increased abundance of granzyme A and granzyme B was observed in culture supernatants of NK cells isolated from pulmonary TB patients with cavity and cocultured with MTB protein lysates.

### 3.3. Cytotoxicity of NK Cells against MTB H37Rv-Infected Monocytes

**Figure 3:**
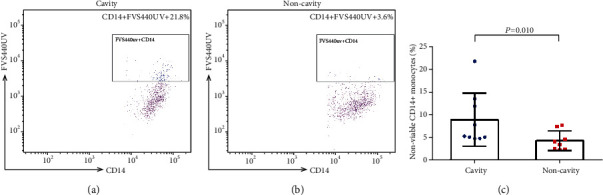
Cytotoxicity of NK cells against MTB H37Rv-infected monocytes. Freshly isolated NK cells from nine pulmonary TB patients with cavity and eight pulmonary TB patients with noncavity were cocultured with MTB H37Rv-infected monocytes to analyze the ability of cytotoxicity. Representative dot plots show the proportion of nonviable CD14+ monocytes infected with MTB H37Rv after coculture with NK cells isolated from pulmonary TB patients with (a) cavity or (b) not. (c) Comparison of the proportion of nonviable MTB H37Rv-infected CD14+ monocytes after coculture with NK cells from pulmonary TB patients with cavity or not. *P* value was derived using a Mann–Whitney *U* test.

As NK cells can release cytotoxic granule contents such as granzyme A, granzyme B, and perforin to trigger programmed cell death and kill intracellular pathogens [18], NK cells play an important role in TB progression. Flow cytometry analysis (Figure 3) showed that increased nonviable MTB-infected CD14+ monocytes were observed after coculture with MTB protein lysates treated NK cells which were isolated from pulmonary TB patients with cavity (8.99 ± 5.82) when compared with those from patients without cavity (4.43 ± 2.15, *P* = 0.01). The results indicate that NK cells from TB patients with pulmonary cavity may have more potent cytotoxic ability to trigger MTB-infected monocyte cell death.

## 4. Discussion

**Figure 4:**
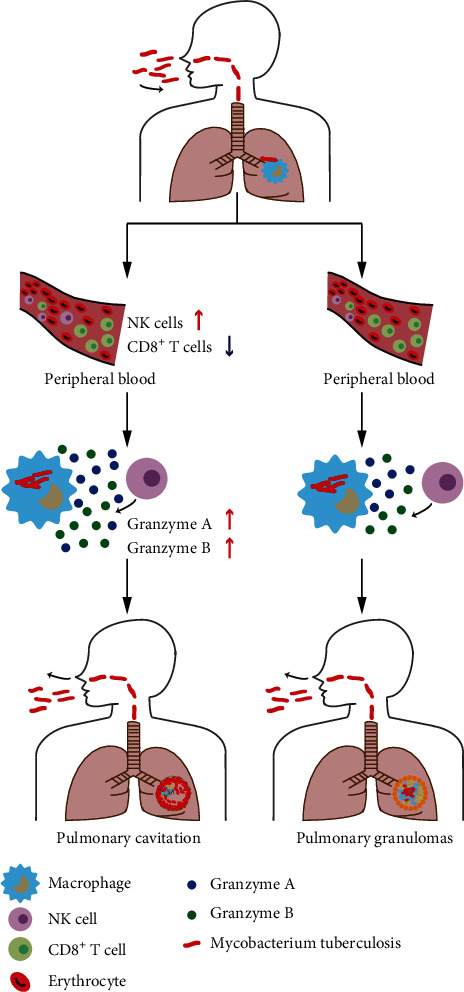
A schematic of the role of granzyme A and granzyme B secreted by NK cells in pulmonary cavitation of TB patients. After infection with MTB, a group of patients have increased proportion of NK cells and decreased cytotoxic CD8+ T cells in peripheral blood. Meanwhile, granzyme A and granzyme B secreted by NK cells during disease progression are increased, which contribute the final pulmonary cavitation.

Despite innate immunity plays a major role in the host's response to MTB infection, more attention has been paid to macrophages that are recognized as the first line of defense to control multiplication of MTB [20]. In this study, our data firstly demonstrated that NK cells participate in cavity formation in pulmonary TB patients (Figure 4). On the one hand, pulmonary TB patients with cavitation had elevated NK cell levels compared with those without cavitation. Early studies suggested that NK cells rapidly expand as a consequence of viral infection. Similar results were observed animal model in response to MTB infection, suggesting an important role for NK cells in controlling intracellular multiplication of tubercle bacilli. Current evidence demonstrates that the vast majority of NK cells in the lung tissue are circulating between the organ and peripheral blood [21]. Thus, the increased peripheral NK cell levels in cavitary patients could likely contribute not only to host protection but also to adverse effects in host tissue. This increase may reflect a higher output of new NK cells from the bone marrow and/or a high proportion of long-lived NK cells. There is strong evidence that interleukin-15 (IL-15) acts as an important cytokine not only to trigger NK cell proliferation but also to promote the survival of proliferating NK cells [22]. Thus, further studies are warranted to elucidate the potential effect of IL-15 on the NK cell homeostasis in pulmonary TB patients with cavity.

On the other hand, the increased cytotoxicity was noted in peripheral NK cells from cavitary patients. Despite traditionally NK cells being considered as nonspecific components of innate immunity, this suggests an MTB-specific NK cell response, which may accelerate the immune activation against tubercle bacilli. This hypothesis is supported experimentally in the animal models that robust antigen-specific NK cell can be identified after both infection and vaccination [23, 24]. Of note, the NK cells from these cavitary donors could induce higher expression of granzymes A and B rather than perforin upon stimulation with MTB antigens, which in turn promotes apoptotic cell death. It has long been recognized that perforin is essential for enabling granzyme-induced apoptosis. We speculate that the high transcriptional level of perforin at baseline warrants entrance of granzymes into targeted cells.

Another interesting finding of our study was that the CD8+ T cell level of cavitary patients was significantly lower than that of noncavitary patients. The low level of CD8+ T cells by flow cytometry suggested one possible interpretation that these peripheral immune cells may be recruited to the tissues of higher antigen concentrations. Early studies have described an important role for the CD4+ T cell in the pathogenesis of cavitation [13, 14]. Decreased CD4+ lymphocytes were reported in patients with cavitary TB compared with noncavitary TB [13]. However, this dogma has been challenged by findings in patients coinfected with HIV that the deficiency of CD4+ T cells is accompanied by a low frequency of cavitary disease [25]. In addition, the comparative studies in animal models confirmed that the mice with little measurable hypersensitivity response do not form true caseous necrosis and cavitation in response to MTB infection. Taken together, the data from HIV patients and animal models suggests that the potent host immune response is essential for formulation of cavitary diseases, although the exact role of adaptive immunologic triggers and cascades are yet to be fully examined.

Our findings have important hints for clinical management of pulmonary TB patients to reduce the occurrence of cavitation. The disease severity of pulmonary TB is not only caused by increasing bacterial load, but also to hypersensitivity to MTB antigens. The excessive activation of NK cells increases the risk of cavitary TB diseases. The multiplication of peripheral NK cells could be used as a surrogate maker for predicting this stage of disease. In addition, given the two-edged sword role of NK cells in TB pathogenesis, the delicate regulation of their function which is aimed at limiting the adverse overactivation of the immune system is necessary after elimination of intracellular tubercle bacilli. Monitoring NK cell dynamics will provide additional benefit in subsets of pulmonary TB patients with early-stage high-risk cavitation.

Our study suffered from several obvious limitations. First, despite enrollment of a large number of patients for determining immune cell composition, the cross-section design hampered the detailed analysis of the effect of disease duration on cavity formation, which majorly caused the lack of correlation between cavitation size and NK cell level. Second, only peripheral blood rather than bronchial lavage fluid was explored. Our findings thus require further validation by analysis of lymphoid compartments of bronchial lavage fluid. Third, despite exploring the transcriptional levels of the major activation receptors for NK cells, no significant difference was noted between TB patients with cavity or noncavity. Further experiments are urgently required to elucidate the molecular mechanisms that trigger human NK cell-mediated cytotoxicity. Fourth, previous experimental data demonstrated that the virulence of MTB could influence the expansion of the NK cell population, which was not taken into consideration in our analysis. Finally, Th1/Th2 homeostasis is an important immune factor to protect host from MTB infection. Thus, it is worth immunophenotyping T helper cell subsets to investigate whether the percentage of Th1 or Th2 cells changed in the TB patients with cavity although the overall percentage helper T cells remained unchanged.

In conclusion, our data firstly demonstrates that NK cells participate in cavity formation in pulmonary TB patients. The elevated NK cell level and increased cytotoxicity of NK cells accelerate the cavitary formulation. These results underscore the need for immunotherapy interventions to limit the adverse overactivation of NK cells. Monitoring NK cell dynamics will provide additional benefit in subsets of pulmonary TB patients with early-stage high-risk cavitation.

## Figures and Tables

**Table 1:** The characteristics of enrolled pulmonary TB patients with cavity or not and healthy uninfected donors.

Characteristics	TB patients with cavity or not		Health control	*P* value
	Yes (*n* = 68)	No (*n* = 61)	(*n* = 20)	
Age (years, mean ± SD)	39.4 ± 18.7	39.6 ± 20.9	39.0 ± 9.9	0.607
Sex				
Male	52	34	11	0.028
Female	16	27	9	
Ethic group				
Han	64	55	20	0.292
Others	4	6	0	
Treatment history				
Primary treatment	38	38		0.460
Retreatment	30	23		

SD: standard deviation. *P* value was derived using the chi-square test.

## Data Availability

The data used to support the findings of this study are included within the article.
